# Are Animals Autistic Savants?

**DOI:** 10.1371/journal.pbio.0060042

**Published:** 2008-02-19

**Authors:** Giorgio Vallortigara, Allan Snyder, Gisela Kaplan, Patrick Bateson, Nicola S Clayton, Lesley J Rogers

## Abstract

Do animals have privileged access to lower level sensory information before it is packaged into concepts, as it has been argued for autistic savants?

Similarities in behaviour between autistic savants and animals have been suggested, most notably by Temple Grandin [1] in her book *Animals in Translation* 2005, and this theory seems to have gained some consensus among other neuroscientists who are not specialists in animal cognition. Here we aim to discuss two specific parallels between the behavioural characteristics of animals and those of autistic savants that have been raised in relation to Grandin's book. Autistic savants show extraordinary skills, particularly in music, mathematics, and drawing. Do animals sometimes show forms of extreme (though, of course, different) cognitive skills confined to particular domains that resemble those shown by autistic savants? We argue that the extraordinary cognitive feats shown by some animal species can be better understood as adaptive specialisations that bear little, if any, relationship to the unusual skills shown by savants. It has also been argued that autistic savants “think in detail”, and that this is the key to their extraordinary skills. Do animals have privileged access to lower level sensory information before it is packaged into concepts, as has been argued for autistic humans, or do they process sensory inputs according to rules that pre-empt or filter what is perceived even at the lowest levels of sensory processing? We argue that animals, like nonautistic humans, process sensory information according to rules, and that this manner of processing is a specialised feature of the left hemisphere of the brain in both humans and nonhuman animals. Hence, we disagree with the claim that animals are similar to autistic savants. However, we discuss the possibility that manipulations that suppress activity of the left hemisphere and enhance control by the right hemisphere shift attention to the details of individual stimuli, as opposed to categories and higher-level concepts, and can thereby make performance more savant-like in both humans and animals. **(Editors note: See Box 1 for Grandin's response.)**

**Box 1. Response by Temple Grandin to the Essay “Are Animals Autistic Savants?”**

*Editors note: We asked Temple Grandin to respond to the essay “Are Animals Autistic Savants?”, which presents a critique, based on studies of comparative animal cognition, of Grandin's premise that animals and autistic savants share similarities in cognition*.

The Essay “Are Animals Autistic Savants?” by Giorgio Vallortigara et al. provides a fascinating overview of the most recent research on animal cognition. I think the basic disagreement between the authors and me arises from the concept of details—specifically how details are perceived by humans, who think in language, compared with animals, who think in sensory-based data. Since animals do not have verbal language, they have to store memories as pictures, sounds, or other sensory impressions. Sensory-based information by its very nature is more detailed than word-based memories. As a person with autism, all my thoughts are in photo-realistic pictures. I can search my own brain, like using Google, for images. As I read about the cognition experiments, I saw the birds performing in my imagination like a virtual reality computer system. The main similarity between animal thought and my thought is the lack of verbal language. Verbal language narrates the images when I “surf the Internet” inside my own brain. If you give me a “keyword,” such as “peanut,” I start to see images like a series of slides shown one after the other. The first image was the Planter's Peanut logo, the second was a Western restaurant that serves peanuts, and the third was a bag of peanuts on a plane.

The Essay by Vallortigara et al. clearly showed that cognition is very real in animals. In normal humans, higher brain processes cover up the sensory-based processing that we share with animals. In *Animals in Translation*, I discussed the work of Bruce Miller, who studied patients with frontal-temporal lobe dementia [1,2]. As the disease destroyed higher brain function, art and music talent emerged. The sensory-based, more-detailed thinking we share with animals was unmasked.

I was most interested to learn that the skills that taxi drivers had developed to navigate by using land markers “had come at the price of acquiring new visual spatial information” according to the authors. Further experiments need to be done with birds to either confirm or disprove Vallortigara et al.'s hypothesis that birds such as the Clark's nutcracker, which has savant-like memory for food storage, has retained good cognition in other domains. My hypothesis is that birds that have savant-like skills for food storage sites or remembering migration routes may be less flexible in their cognition. It is well known that people with autism do poorly on the Wisconsin card sorting task, where colors and shapes have to be sorted into different categories. The person with autism is slower to respond correctly when the category is switched from a shape to a color.

An operant conditioning task could be used to compare flexible problem solving in migratory and food-storing bird species to species that do not have savant-like skills. The task could be to peck a lever when a light comes on. After the bird had learned this, the stimulus would be switched to a sound. I predict that the more savant-like birds will require more trials to switch back and forth between the light and the sound cue.

I am pleased that my book has stimulated so much discussion, and I hope it stimulates more research on animal cognition.

Temple Grandin is the author of *Animals in Translation* and is Professor of Animal Science, Colorado State University, Fort Collins, Colorado, United States of America. E-mail: Cheryl.miller@colostate.edu

**References**

1. Miller BL, Boone K, Cummings JL, Read SL, Mishkin F (2000) Functional correlates of musical and visual ability in frontal temporal dementia. Br J Psych 176:458-463.

2. Miller BL, Cummings J, Mishkin F, Boone K, Prince F, et al. (1998) Emergence of art talent in frontal temporal dementia. Neurology51:978-981.

## Do Animals Think like Autistic Savants?

When Grandin proposed similarities in cognition between autistic savants and animals, she reasoned on the basis that animals, like autistic humans, sense and respond to stimuli that nonautistic humans usually overlook. In other words, animals respond to and remember the details of the world around them, whereas nonautistic humans overlook the details in favour of the overall whole. Since Grandin's book *Animals in Translation* shows extraordinary insight into both autism in humans and animal welfare, the question deserves scrutiny from scientists working on animal cognition and comparative neuroscience.

When Grandin proposed similarities in cognition by autistic savants and animals, she reasoned on the basis that animals, like autistic humans, sense and respond to stimuli that nonautistic humans usually overlook.

Put in terms of pathology, the question does not make sense, and Grandin was not referring to animal models that are used to study autism (e.g., rodents treated with pharmacological agents or raised in restricted contexts that induce repetitive behaviour [2,3]). She was referring to the animals she has studied, such as farm animals and domestic pets. Since many of these animals experience unique physical and psychological conditions that can be stressful and even distressing, their mental processes may be quite different from those of wild animals. We wish to address the question of animals as autistic savants in the broad context of animal behaviour in the natural environment.

A possible relationship between the thinking of autistics and that of nonhuman animals has been suggested with respect to the so-called “theory of mind”, i.e., the ability of members of our species to attribute intentional (mental) states to others, and its absence or weaker expression in both autistics and animals. Whether nonhuman animals have a fully developed theory of mind is still a controversial issue (for a review, see [4]) and one with which we are not specifically concerned here. However, impaired social behaviour is an aspect of autism in humans that is not shared by social, nonhuman animals.

More recently, autism has been linked to a possible deficit in the functioning of mirror neurons in the cortex [5,6]. Because mirror neurons are active in matching actions performed by others, even when those actions are merely observed, they are seen as “a potential bridge between minds” [6] and certainly as essential to social behaviour. Since we know that at least some nonhuman species have mirror neurons (e.g., macaques [7]), the proposed impairment of mirror neurons as a core aspect of autism in humans provides no support for the hypothesis that nonhuman animals may be autistic savants, at least as a general characteristic of a species. This does not preclude the possibility that, within a species, individual animals with impaired mirror neuron functioning could be autistic.

Savant abilities come at a cost in other aspects of processing and, therefore, appear to be unrelated to the extraordinary species-specific adaptations seen in some taxa.

Snyder and Mitchell [8] consider the salient aspect of autistic savants to be their “privileged access” to lower-level sensory information before it is packaged into holistic concepts and labels (see also [9]). Accordingly, the mental processes of autistic humans, which are less governed by rules and learned concepts that pre-empt and filter the perceptions of nonautistic humans, are more literal and less categorising than those of nonautistics. This type of mental processing is often seen as lower-order processing and hence as a possible link to the mental processing abilities of nonhuman animals. It is this interpretation that we shall address.

## Extraordinary Cognitive Skills

Certain animal species do show extraordinary skills in perceptual and motor behaviour when compared with humans or other animal species. Echolocation of bats or dolphins or the motor performance of a hunting feline are just two examples of such extraordinary abilities; yet such skills could be better characterized as adaptive specialisations. The same is probably true in a more-cognitive domain. Food-storing birds show impressive memory capabilities (reviewed in [10]). Clark's nutcrackers, for example, can store and remember thousands of caches in the wild [11]. Specific neurological adaptations accompany these extraordinary behaviours: the hippocampus of food-storing birds is enlarged compared with that of phylogenetically related species of birds that have not developed caching abilities (reviewed in [12]). Other studies have shown that the hippocampal enlargement in food storers arises as a result of the specific memory demands required for accurately recovering previously cached food [13,14]. As in the case of autistic savants, the extraordinary cognitive abilities of Clark's nutcrackers are limited to a specific domain (that of spatial cognition). However, unlike autistic savants, Clark's nutcrackers do not show any impairment in other cognitive domains. The birds' performance when using object-specific cues (as opposed to their extraordinary ability when using spatial-specific cues) is most likely the same as that shown by non–food-storing species, and comparable to the performance of ordinary avian species [12]. This contrasts to autistic savants, who have impaired cognitive abilities in domains other than the one(s) in which they show extraordinary abilities [15,16]. In other words, the neurological adaptation in food-storing birds—the enlargement of their hippocampus—does not seem to be at the expense of reduced neural tissue in other parts of the brain [17]. It must be said, however, that it may be difficult to locate a compensatory reduction of another brain region, and no research has focused on discovering this.

Interestingly, domain-specific cognitive specialisations that result from extensive training do show some form of neurological compensation: it has been reported that, in licensed London taxi drivers, an increase in the volume of the posterior hippocampus is associated with a relative decrease in the anterior portion of the same structure [18]. Fascinatingly, when London taxi drivers were compared with London bus drivers, matched for driving experience and levels of stress but differing in that the latter had to follow a constrained set of routes, the taxi drivers were found to show better scores than the bus drivers on London landmarks and proximity tests; however, the ability to acquire new visuo-spatial information was poorer in taxi drivers than in bus drivers, apparently as a result of their showing a reduced anterior part of the hippocampus [19]. The extraordinary ability to navigate using landmarks acquired by the taxi drivers had come at a price—acquiring new visuo-spatial information—unlike the extraordinary cognitive adaptation of spatial memory in the Clark's nutcracker, which was an addition without expense. Savant abilities come at a cost to other aspects of processing and, therefore, appear to be unrelated to the extraordinary species-specific adaptations seen in some taxa.

Certain animal species can reproduce sounds from sources other than those that are species-specific, and do so in an extraordinarily detailed fashion. In songbirds, this ability to memorize and reproduce sounds depends on a set of nuclei in the forebrain [20]. For most humans without any impairment, the ability to reproduce sounds tends to be limited to some phrases. Usually, it requires practice and repeated exposure to reproduce a whole song or musical passage, and to produce a complete rendition without such repetition is rare. Mozart was portrayed in the film “Amadeus” as a musical savant because he was able to write down an entire concerto on the basis of having heard it once. Some birds may be able to compete with this ability. Australian magpies have been recorded as mimicking a complex sequence of sounds from a kookaburra duet or even learning a whole song on a single exposure [21]. Sonograms have revealed that the structure of the rendering was also extremely accurate. Whether or not one can compare Mozart's achievement with that of a bird remains questionable, but it appears that the principle in each case is the same: the entirety of the sound sequence had been absorbed, encoded, and committed to memory well enough that its faithful reproduction was possible. It is not known in the case of magpies whether their ability leads to compensatory diminishment of other areas in the brain. Their song system is the same as in other songbirds [22], and their hyperpallium (referring to visual processing) is, in fact, especially large [23,24].

## Do Animals Think in Details?

It has been argued that autistic people think in details and have difficulty grouping attributes into holistic pictures and forming labels. One autistic boy, for example, learned the concept of a giraffe through a detail—the pattern of the coat—not the long neck or overall shape. As a result, he also identified a leopard as a giraffe [15,25–27].

Grandin states animals think like autistic savants. Normal humans are good at seeing the big picture but bad at what Grandin calls “all the tiny little details that go into that picture”. For normal humans, the big picture is not created by accumulating sensory details, but rather by filtering out details [27]. Like autistic people—and unlike normal humans—animals, Grandin argues (as does Snyder for autistic savants) have direct access to the raw sensory data that an ordinary human brain would discard by filtering out. Grandin also argues that humans evolved larger association areas than nonhuman animals and that, in order to avoid overloading these association areas, access to raw sensory data should be strictly limited.

Very generally, the left hemisphere sets up rules based on experience and the right hemisphere avoids rules in order to detect details and unique features that allow it to decide what is familiar and what is novel. This is true for human and nonhuman animals, likely reflecting ancient evolutionary origins of the underlying brain mechanisms.

We believe, however, that there is little evidence in support of these views. It is true that when humans process visual stimuli, global information often takes precedence over local information. For instance, in Navon's experiments [28], people are shown collections of a particular letter arranged to form a global configuration that is also a letter (e.g., an “H” made up of “S”s). Identification responses are usually faster for the global configurations than for the local letters. Some studies have pointed to a local precedence effect in both pigeons [29] and nonhuman primates [30–32]. However, more recent work has shown that local information does not always take precedence over global information in nonhuman animals. For instance, in certain conditions, pigeons can acquire a global-feature categorisation more rapidly than a local-feature categorisation, exactly as do humans [33]. It seems that the local-feature precedence observed in these studies simply results from the specific stimuli and procedures employed (e.g., the subtended visual angle and the density of local elements, see also [34]).

Similar arguments apply to the celebrated studies (e.g., [35]) showing that pigeons showed no decrement in recognizing stimuli that were spatially scrambled, and therefore suggesting that pigeons' discriminative responding is controlled by local features alone. In contrast, more recent work has shown that pigeons use both global and local aspects of the stimuli, with different mixtures of these types of information depending on the particular perceptual context [36]. For instance, pigeons trained to discriminate cartoon people on a variety of background scenes attend to global aspects of cartoon people; on the other hand, using line drawings of cartoon faces as stimuli and examining the effects of scrambling and deletion of components showed that a set of components (eyes and eyebrows) appear to exert strong control over behaviour and scrambling only moderately suppresses responding [37].

The mechanisms that determine prevalence of processing details or the big picture seem to be similar in human and nonhuman species. There could be two senses in which “details” are assembled into a big picture. One is the operating of preattentive mechanisms of perceptual grouping to segregate figures from ground and parse objects in visual scenes. These mechanisms are largely bottom-up and data driven. The other is the extraction, from already segregated and parsed details and parts of stimuli and events, of higher-order categories and concepts on the basis of past experience. These mechanisms are attentive and affected by overall knowledge and beliefs. They are top-down mechanisms.

As to the early processing mechanisms of segregation and grouping, here sometimes differences between species can be observed, and certain animals may indeed show an inclination to attend to details in some contexts, probably because of specific adaptations. Pigeons, for instance, when faced with illusory figures like the famous Kanizsa's triangle [38] (Figure 1) show a tendency to attend to the inducing pacman rather than extracting the overall triangular figure.

**Figure 1:**
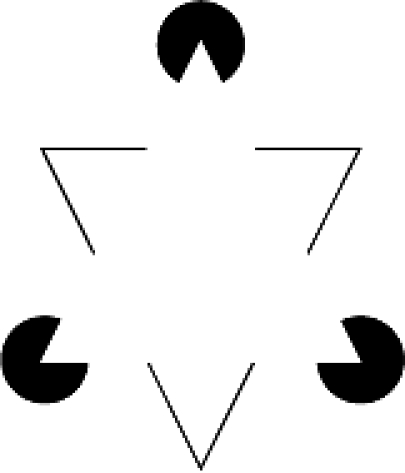
The Kanizsa Triangle Animals do experience the illusion, although some species may be more inclined to attend to details, of the pacman before extracting the overall triangular shape (see text).

In a study on subjective contours with Kanizsa's triangles and squares, Prior and Güntürkün [39] found that only four out of 14 animals tested succeeded. As indicated by control tests, pigeons responding to subjective contours were attending to the global pattern of the stimuli, whereas pigeons not responding to subjective contours were attending to the local details of the stimuli. Pigeons also showed similar difficulties when dealing with recognition of partly occluded objects, to which they tend to respond literally to the visible parts instead of mentally completing them [40,41]. It is important to note, however, that this clearly shows relative preference for pigeons to attend to some aspects of a visual scene rather than an absolute inability, because more recent evidence has revealed that pigeons are able both to see subjective contours [42] and to complete partly occluded objects [43,44]. Also, this is clearly not a general characteristic of the avian brain as opposed to the mammalian brain, because other species of birds show no attributes similar to those of pigeons (e.g., domestic chicks, zebra finches and owls; see [45–49]).

It seems likely that there could be ecological differences favouring perception based on response to parts or reconstruction of whole objects on the basis of interpolation between these parts. Linking function to structure, it is interesting to observe that in experiments using conditioning procedures, such as those performed with pigeons, the stimuli were presented in the frontal binocular visual field of the animals, a portion of the visual field that is mainly represented within the tectofugal pathway in pigeons [50,51]. The frontal visual field seems to be specialized for (myopic) foraging for food on the ground, whereas the lateral visual field seems to be specialized for predator detection and flight control. Near-sighted acuity would favour examination of fine stimulus details (as demonstrated in use of the frontal, binocular field in selecting between food types [52]) and may be responsible for the local advantages observed in most experiments that used frontal presentations of visual stimuli. The lateral visual fields, in contrast, may be more concerned with the larger scale integration of scene and flight control information [53], thus showing more sensitivity to global information (see [54]).

Specialisation of the left and right sides of the brain (lateralisation) may be instrumental in attending to detail or the big picture in the first of the two senses mentioned above. As in humans [55–57], gestalt-like properties of visual scenes seem to be associated with right hemispheric function in animals (reviewed in [58–60]. In chicks, amodal completion has been proven to be more likely to occur under right hemispheric control [61], exactly as it is in humans [62]. Moreover, the right hemisphere of the chick attends to global, spatial cues as opposed to landmark cues, to which the left hemisphere attends [63].

There is however another, more top-down, route to the integration of details for the formation of a big picture: a specialisation of the left hemisphere. We see what we expect. And what we expect is encapsulated in our mindsets—mental templates formed largely by experience. Brains are wired to present us with the best hypothesis, the best guess based on our prior experience, especially experience in early life [27,64]. But autistic savants, according to some conceptions, would be literal in the sense that, overwhelmed by details, they would be impaired in their ability to extract general, higher-order concepts on the basis of past experience. This means that they would have less access than nonautistics to left hemisphere processing.

Animals, of course, cannot be autistic in this sense. They are not literal, but have mindsets allowing them to be fast at executing their lifestyle within their particular niche. Unlike autistic people, animals are not overwhelmed by detail. Quite interestingly, they also show brain specialisations similar to those revealed in humans in terms of the roles that the two cerebral hemispheres play in assembling categories and thus discarding idiosyncratic details in favour of invariant properties when analyzing stimuli and events (reviewed in [58,65–67]).

The savant syndrome is often associated with some left brain dysfunction, together with right brain compensation [68]. Snyder has shown [69–73] that it is possible to induce savant-like skills in normal healthy people by inhibiting the left anterior temporal lobe with magnetic pulses at the rate of 1 pulse per second—a process known as transcranial magnetic stimulation, or TMS. But why does this inhibition of the left anterior temporal lobe induce savant-like skills?

Gazzaniga [74] refers to the function of the left hemisphere as the “interpreter”. The left hemisphere tends to create a belief system or mindset and to fold any new information into the existing belief system. The hemispheres are in competition with one another, with the hypothesis-imposing left hemisphere dominating or inhibiting the right hemisphere, which is more open to novelty [27]. The right hemisphere, on the other hand, tends to play, as Ramachandran [75] has put it, the role of the “Devil's advocate”, looking for novelty and inconsistencies. Because of its duty to recognize novelty, the right hemisphere is forced to maintain a more literal record of the events.

It is quite fascinating that similar specialisations of the two hemispheres have been reported for the nonhuman animal brain (reviewed in [76]). In the domestic chick, for example, which is probably the most investigated animal species with respect to brain lateralisation [65,77,78], evidence has been collected that the right hemisphere mainly attends to novelty and focuses on those unique and idiosyncratic properties of visual stimuli that may help to establish whether a particular stimulus is or is not the same as the one encountered before. The left hemisphere, in contrast, tends to attribute stimuli to categories in order to provide fast and efficient responses. In so doing, the left hemisphere tends to attend to the general characteristics of the stimuli, rather than to the details (see Figure 2). (Note that by attention to “details” here, we do not mean raw sensory information, but rather the information on parts available after applying gestalt mechanisms of perceptual processing at the preattentive level.) A noticeable example is the role of the two hemispheres in social recognition associated with imprinting, in which the left hemisphere builds up a representation of the general properties of the social partner (without including in it individual recognition), whereas the right hemisphere encodes the peculiar and unique properties of a social partner as an individual [79, 80]. Similarly, when presented with grains scattered on a background of inedible pebbles, a chick using its left hemisphere categorises the array of stimuli as food/nonfood and avoids pecking at the pebbles, whereas a chick using the right hemisphere pecks at both grain and pebbles, each as a separate and novel entity [81].

**Figure 2:**
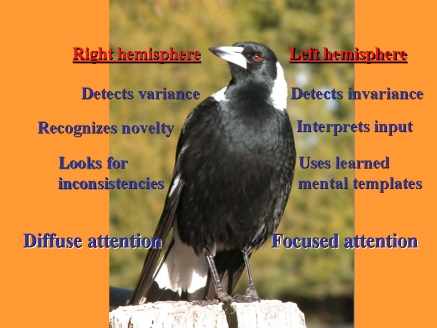
Lateralization in the Avian Brain, Illustrated Using the Australian Magpie, Gymnorhina tibicen Behaviour controlled by the left and right hemispheres is listed. As in humans, functions of the left hemisphere are consistent with those of autistic humans. (Photograph: G. Kaplan, Centre for Neuroscience and Animal Behaviour, University of New England, Australia)

A general theoretical framework for these findings has been proposed, based on the idea of “functional incompatibility” [67]. When assessing a novel stimulus—an event likely to be faced quite commonly even by the most primitive vertebrates—an organism must carry out two different types of analyses. First, it must rely on previous comparable experiences to estimate the degree of novelty of the stimulus, and to do so it must recall stored memories and then elaborate on them for future use. Secondly, certain appropriate cues, based on past experience (or on phylogenetically based information), must be used to try to assign the stimulus to a category, and so to decide what sort of response (if any) should be given. Categorisation must be made on the basis of selected stimulus properties, despite variation in many other properties. All this is reminiscent of the functional incompatibility among logical demands, a condition hypothesized to underlie the evolution of multiple memory systems by Sherry and Schachter [82]. To categorise events (or stimuli), the organism must recognise, and memorise, those features of an experience (or of a stimulus) that recur in different episodes (or stimuli) and, at the same time, ignore or discard unique and idiosyncratic features that do not recur and thus are not essential to learning. The selective attention that results is one of the brain's main functions, as it enables the smooth, and eventually automatic, execution of skilled motor behaviour, performed in response to certain invariant features of episodes. In contrast, to detect novelty and to build up a detailed record of episodic experiences, the organism must attend to the contextual details that mark individual experiences uniquely, i.e., to recognize variance across episodes rather than invariance. Processing of information about invariance and variance between experiences requires the use of mutually incompatible processes that might best be handled by functionally separate systems.

Overall, and very generally, the left hemisphere sets up rules based on experience, and the right hemisphere avoids rules in order to detect details and unique features that allow it to decide what is familiar and what is novel. This is true for human and nonhuman animals, likely reflecting ancient evolutionary origins of the underlying brain mechanisms.

None of the evidence mentioned above favours the idea that animals attend to details more than humans do. The balance between attending to details or the big picture may, of course, vary between species and within the same species depending on ecological constraints and perceptual contexts, but it is performed by similar brain mechanisms, without any clear-cut divide between human and nonhuman species.
